# Field responses of *Glossina pallidipes* and *Glossina fuscipes fuscipes* tsetse flies to Novel Repellent Blend and Waterbuck Repellent Compounds in Kenya

**DOI:** 10.1371/journal.pntd.0013367

**Published:** 2025-07-28

**Authors:** Benson M. Wachira, Richard Echodu, Johnson O. Ouma, Imna I. Malele, Daniel Gamba, Michael Okal, Kennedy O. Ogolla, Clement Mangwiro, Robert Opiro, Deusdedit J. Malulu, Bernard Ochieng, Raymond E. Mdachi, Paul O. Mireji

**Affiliations:** 1 Biotechnology Research Institute, Kenya Agricultural and Livestock Research Organization, Kikuyu, Kenya; 2 Department of Chemistry, Pwani University, Kilifi, Kenya; 3 Department of Biology, Gulu University, Gulu, Uganda; 4 Vector Health International, Africa Technical Research Centre, Arusha, Tanzania; 5 Vector and Vector Borne Diseases Institute, Tanzania Veterinary Laboratory Agency, Tanga, Tanzania; 6 Technical Department, Kenya Tsetse and Trypanosomiasis Eradication Council (KENTTEC), Nairobi, Kenya; 7 Department of Animal Health, International Centre for Insect Physiology and Ecology, Nairobi, Kenya; 8 Department of Animal Science, Bindura University of Science Education, Bindura. Zimbabwe; 9 Wildlife Research and Training Institute, Kwale, Kenya; Institut Pasteur, FRANCE

## Abstract

Control of tsetse flies constitutes a cornerstone of trypanosomiasis control and elimination efforts in Africa. The use of eco-friendly odor-based bait technologies has been identified as a safer method for control of tsetse flies. These technologies are significantly augmented by development of effective repellents that reduce contact between trypanosome-infected tsetse flies and their vertebrate hosts. Waterbuck Repellent Compounds (WRC) and Novel Repellent Blend (NRB) are recently developed tsetse fly repellent formulations. Information on relative efficacy of these formulations against major tsetse fly vectors of trypanosomiasis in Kenya is limited. Such information can inform choices of repellent technology for optimal control of the flies. Here we assessed relative field responses of *Glossina pallidipes* and *G. fuscipes fuscipes*, representative of savannah (morsitans) and riverine (palpalis) groups of tsetse flies, respectively. We deployed NG2G traps or sticky panels and tiny targets using randomized Latin Square experimental design. We then assessed catches of *G. pallidipes* or *G. f. fuscipes* respectively on the traps/panels in the absence or presence of WRC or NRB. We additionally baited the NG2G traps with *G. pallidipes-*responsive 3-propylphenol, octenol, p-cresol, and acetone (POCA) attractant blend, that effectively served as proxy for the preferred vertebrate natural host. We performed the *G. pallidipes* and *G. f. fuscipes* experiments in Shimba Hills National Reserve and Ndere Island National Park respectively in Kenya and incorporated a no-odor control for each set of experiments. Mean catches of male *G. pallidipes* in traps without odor (control), baited with POCA, POCA with WRC or POCA with NRB were 9.86 (95% CI; 6.50- 14.74), 42.71 (95% CI; 28.11 - 64.62), 14.30 (95% CI; 8.50 - 23.60) and 3.03 (95% CI; 0.89 - 7.59) respectively, while for females, the catches were 24.43 (95% CI; 13.65 - 47.42), 70.93 (95% CI; 42.95 - 120.50), 23.85 (95% CI; 16.33 - 37.84) and 6.82 (95% CI; 3.59 -17.02) flies per trap per day respectively. Consequently, the NRB was 4.72 and 3.50-folds and significantly (P < 0.001) more repellent to male and female *G. pallidipes* respectively, than WRC. In contrast, catches of *G. f. fuscipes* on targets were similar (P > 0.05) across all the three treatments (including no-odor control). The NRB and WRC are thus efficacious against *G. pallidipes* but not *G. f. fuscipes*, with efficacy of NRB being several-folds that of WRC against *G. pallidipes.* Whether these profiles represent general responses of morsitans and palpalis group of tsetse flies remains to be determined. Additionally, *G. f. fuscipes* merits further research to formulate an effective repellent against this fly. The NRB can potentially provide better protection to vertebrate hosts, including humans and their livestock than WRC from *G. pallidipes*. Consequently, NRB can be integrated into routine trypanosomiasis control program to stem transmission of trypanosomes by *G. pallidipes*, especially in eastern and southern Africa where *G. pallidipes* is naturally abundant.

## Author summary

Tsetse flies are sole biological vectors of Human African Trypanosomiasis (HAT) and Animal African Trypanosomosis (AAT) in sub-Sahara Africa. Tsetse fly repellent technologies that reduce contact between tsetse flies and their vertebrate hosts can disrupt transmission cycle of HAT and AAT. Waterbuck Repellent Compounds (WRC) and Novel Repellent Blend (NRB) are recently developed tsetse fly repellent formulations. Information on relative efficacy of these formulations against major tsetse fly in Kenya is limited. Such information can inform choices of repellent technology for optimal control of the flies. We assessed relative field responses of *Glossina pallidipes* and *Glossina fuscipes fuscipes*, representative of savannah (morsitans) and riverine (palpalis) groups of tsetse flies, respectively, to these repellent formulations. We established that WRC and NRB were effective against *G. pallidipes*, with NRB exhibiting 3.5 and 4.7-folds better repellence of female and male *G. pallidipes* than WRC. None of the repellents was effective against of *G. f. fuscipes*. Whether these profiles represent general responses of morsitans and palpalis group of tsetse flies remains to be determined. The *G. f. fuscipes* merits further research to formulate an effective repellent against this fly. The NRB can potentially better-protect vertebrate than WRC from *G. pallidipes.*

## Introduction

Human African Trypanosomiasis (HAT) and Animal African Trypanosomosis (AAT) are among the most Neglected Tropical Diseases (NTDs) with devastating health and economic consequences in sub-Sahara Africa [1,2]. The AAT is responsible for mortality of about three million cattle and loss of about US$ 4.75 billion per year in terms of agricultural Gross Domestic Product [3]. With no effective vaccines, and limited chemotherapeutic and chemoprophylactic management options against HAT and AAT, control of tsetse flies constitutes a cornerstone in the campaign towards suppression and elimination of trypanosomiasis. Insecticide application to cattle for tsetse control was demonstrated in southern Africa in the mid 1980s, but seemed of limited use where the intention was to remove tsetse flies from spatially wide areas or where cattle were absent or could not be introduced [4]. However, the application is currently considered the most economical method of tsetse fly control [5], with recent application estimated to significantly enhance mortality of the flies by 5–14% daily at the interface of wildlife and livestock in Serengeti, Tanzania [6]. Control of tsetse flies through aerial and ground spraying is limited by detrimental environmental effects of the aerially delivered insecticides on non-target organisms [7]. Alternative eco-friendly bait “pull” technology for tsetse fly control consists of stationary traps and targets that exploits visual and olfactory responses of tsetse flies to their hosts [8]. The traps and targets are typically baited with attractant odor blends that mimics the smell of buffalo (*Syncerus caffer*), a preferred host of most savannah tsetse fly species [9]. The targets are usually impregnated with biodegradable deltamethrin pyrethroid insecticide that effectively kills or knocks-down the flies when they land on the target. This technology has been used successfully to control tsetse flies in environmentally sensitive and diverse forest, riverine and savannah ecosystems due to the relatively high specificity and minimal environmental contamination components [10].

On the other hand, technology of spatial odor repellent of tsetse flies has recently been devised [11,12]. The technology deters tsetse flies from interacting with vertebrate hosts of the flies, such as humans and their livestock, effectively impeding transmission of trypanosomes through infective bites from the flies. This technology complements the bait technology above, by establishing a formidable “push (repellent) – pull (attractant)” technology that can rapidly suppress tsetse fly populations and associated transmission of HAT and AAT causing trypanosomes. Formulation of the spatial repellents was informed by tsetse fly’s (*Glossina pallidipes* or *Glossina morsitans morsitans*) active avoidance behaviors of waterbuck bovid (*Kobus ellipsiprymnus defassa*) vertebrate animals [9,13]. Assessments of chemical composition of the allomones emitted by the waterbuck revealed guaiacol, geranylacetone, pentanoic acid and δ-octalactone as the key components [14]. These components were formulated and field optimized into a Waterbuck Repellent Compound (WRC) blend, comprising of 2:1:3:3 blend of guaiacol, geranylacetone, pentanoic acid and δ-octalactone respectively for effective field repellence of *G. pallidipes* [12]. This formulation provided substantial protection to livestock from tsetse fly transmitted trypanosomes [15]. Follow-up studies on the effects of structural variants (analogs) of δ-octalactone on olfactory/behavioral responses of *G. pallidipes* or *G. m. morsitans* established that replacing δ-octalactone with its δ-nonalactone analog enhanced repellency to both species [16], and combining δ-nonalactone, heptanoic acid, 4-ethyl guaiacol and geranyl acetone in a 6:4:2:1 proportion generated a Novel Repellent Blend (NRB) effective against both tsetse fly species [11]. However, relative efficacy of these repellents (WRC and NRB) against these tsetse fly species and *G. f. fuscipes,* an important vector of HAT in eastern and central Africa, is poorly understood. Such information can inform choice of appropriate repellent for more efficacious protection of humans and their livestock from tsetse fly bites and associated trypanosomiasis transmission. This study was initiated to establish the relative field repellence of *G. pallidipes* and *G. f. fuscipes* by WRC and NRB in Shimba Hills National Reserve and Ndere Island National Park where *G. pallidipes* and *G. f. fuscipes* respectively are naturally abundant.

## Materials and methods

### Study areas

The studies were conducted at Shimba Hill National Reserve (-4°15′26″S, 39°23′16″E; altitude 403 m) in Kwale County along the coast, and Ndere Island National Park (-0°12′22″S, 34°30′44″E; altitude 1,228 m) along the shores of Lake Victoria, in western Kenya. Tsetse fly species in the Shimba Hill National Reserve include *G. pallidipes*, *G. austeni* and *G. brevipalpis* while *G. f. fuscipes* is the only tsetse fly species inhabiting the Ndere Island National Park. The Shimba Hill National Reserve is about 300 Km^2^ and is inhabited by sable antelopes, buffalos, syke monkeys, elephants, giraffes, leopards, genets, waterbucks, African bush babies, civet cats, hyenas, bush pigs, coastal black and white colobus monkeys, red, blue and bush duikers, greater galagoes, black-faced vervet monkeys, bushbucks, serval cats, black and red shrews, knob-bristled suni shrew and many more. The vegetation in the reserve consists mostly of coastal rain forest and semi-evergreen woodland and grassland. The reserve region experiences long and short rainy seasons from April to June, and October to November, respectively, with 855 mm −1682 mm annual rainfall, mean annual temperature of 24.2 °C, with the highest daily temperatures of 33°C in February-March and November, and lowest temperature of 21 °C in July-August. The Ndere Island National Park is a 4.2 km^2^ island and supports a variety of animals that include hippos, monitor lizards, Nile crocodiles, several fish species, snakes, baboons, impalas, the rare sitatunga antelopes, water bucks, zebras and warthogs among others. The island is inhabited by grassland and shrubs, indigenous trees and woodland forest along the shorelines of the lake. The park features a tropical rainforest climate with no true dry season and significant rainfall year-round. Annual rainfall is about 276.22 mm. Mean annual temperature is 22.9°C, with the highest daily temperatures of 30 °C. Approvals to perform experiments in the protected areas were obtained from the Kenya Wildlife Service (KWS)(permit No. KWS/BRM/5001), National Commission for Science, Technology & Innovation (NACOSTI) (permit No. NACOSTI/P/18/28381/22226), and the National Environment Management Authority (NEMA) (permit No. NEMA/AGR/93/2018) in Kenya.

### Test chemical compounds and blends

We sourced for pure (98–99%) δ-nonalactone, δ-octalactone, geranyl acetone, acetone, heptanoic acid, pentanoic acid, 4-ethyl guaiacol, 1-octen-3-ol, 3-n-propylphenol and p-cresol (4-methylphenol) from Sigma-Aldrich, Taufkirchen, Germany. For experiments, we separately formulated 1) WRC blend comprising of guaiacol, geranylacetone, pentanoic acid and δ-octalactone (2:1:3:3) (Saini et al., 2017), 2) NRB comprising of δ-nonalactone, heptanoic acid, 4-ethyl guaiacol and geranyl acetone (6:4:2:1) [11] and 3) POCA, comprising of 3-n-propylphenol (P), octanol (O) and p-cresol (C) (1:4:8), which together with separately released acetone (A), form an established odor blend attractant of *G. pallidipes* [17,18].

### Experimental design

We employed randomized Latin Square Design (LSD) experimental approach in assessment of field responses/catches of adult *G. pallidipes* or *G. f. fuscipes* to the treatments. The responses of *G. pallidipes* and *G. f. fuscipes* were assessed in Shimba Hills National Reserve and Ndere Island National Park, respectively. In assessing the responses of *G. pallidipes* to NRB or WRC, a series of NG2G traps [19] were deployed in the field, at intervals of 300 m to minimize spatial interaction between treatment effects in an LSD experiment comprising of four traps (treatments) in four sites randomly rotated within four days, such that each treatment was deployed on each site once as we previously described [11,16,20]. Treatments comprised of the trap with or without 1) odor treatment (no-odor control) 2) POCA, 3) POCA with WRC or 4) POCA with NRB in three independent replicates, conducted in three separate independent blocks 1–2 km apart within the study area. Consequently, we deployed nine (3X3) independent replicates of each treatment. We dispensed 1) 3-n-propylphenol, octanol and p-cresol (POC), WRC or NRB using sealed thin-walled polythene sachets constructed from polyethylene lay flat tubing (150 microns thick) folded to form a surface area of about 50 cm^2^ [11] and acetone (A) using 28 ml clear glass universal bottles. The sachets were pegged 25 cm above the ground and 30 cm downwind from the center of respective traps, and trapped flies were collected at 24-hr intervals, at 5:00 pm each day, to encompass morning and afternoon bimodal *G. pallidipes* activity peaks [21]. The flies were then identified to species level using standard taxonomic keys as outlined in Pollock [22]. We established the release rates of the odors and documented catches (counts) of the flies (*G. pallidipes*) by sex, treatment, site (trap), day and block. Mean release rates for WRC and NRB were established based on daily loss in masses, pre- vs post- deployment, as 6.45 ± 0.14 and 6.32 ± 0.09 mg/h respectively. POCA bait with two componentsPOC and Acetone had mean release rates of 7.89 ± 1.02 and 495.35 ± 6.11 mg/h, respectively.

We assessed responses of *G. f. fuscipes* to WRC or NRB using the same approach we used in assessing responses to *G. pallidipes* as described above. However, POCA was excluded from the treatments since *G. f. fuscipes* responses are not influenced (non-responsive to) by POCA [23,24]. This effectively reduced the experimental design to three treatments (no-odor control, WRC and NRB) for *G. f. fuscipes*. Additionally, we replaced the NG2G traps [19] with targets (with sticky surface) that have been demonstrated to have better efficacy in trapping *G. f. fuscipes* [25]). Consequently, the LSD experiment comprised of three targets (treatments) in three sites randomly rotated within three days.

### Data analysis

We assessed for differences in patterns of catches between sexes of *G. pallidipes* or *G. f. fuscipes* tsetse flies using Chi-Square, and where the patterns of catches did not significantly differ between the sexes, we pooled data for both sexes for Analysis of Variance (ANOVA), or separately analyzed the data, using ANOVA, where the differences were significant. For ANOVA, we first transformed counts of tsetse fly catches for each treatment and replicate to log (n + 1) to normalize the distributions and homogenize the variances. We used general linear model one-way ANOVA for LSD data with transformed catches as response variable, treatments as factors and sites/days as blocks. We separated the means by Tukey’s Honestly Significant Difference (HSD) post hoc test and back-transformed the data to antilog-1 for reporting the geometric means (± 95% Confidence Intervals) [26]. We obtained catch indices for *G. pallidipes* or *G. f. fuscipes* for different treatments by expressing the response of each treatment category as a proportion of responses to control without odor treatment. We ensured that the transformed data met the normal distribution assumption requirement for t test using Shapiro-Wilk test, and homogeneity of variance requirement for ANOVA using Levene test. Additionally, we assessed for additivity of variance by evaluating the interactions between sites, days and treatments. We summarized descriptive statistics, where necessary, and presented them as means with their ± 95% Confidence Intervals. Analyses were performed using R [27] and GraphPad Prism version 10.0.0 for Mac [28].

## Results

**Fig 1:**
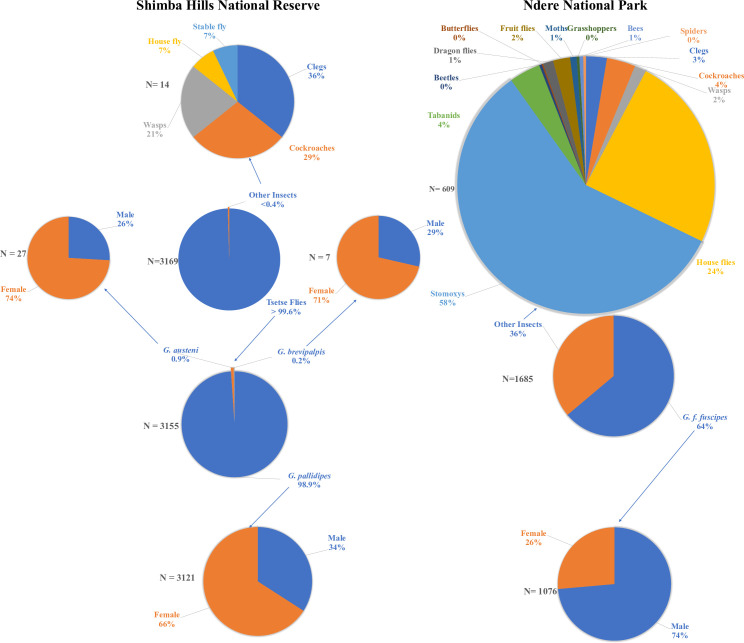
Distribution of catches of arthropods in traps and targets in Shimba Hills National Reserve and Ndere National Park respectively, in Kenya.

**Fig 2:**
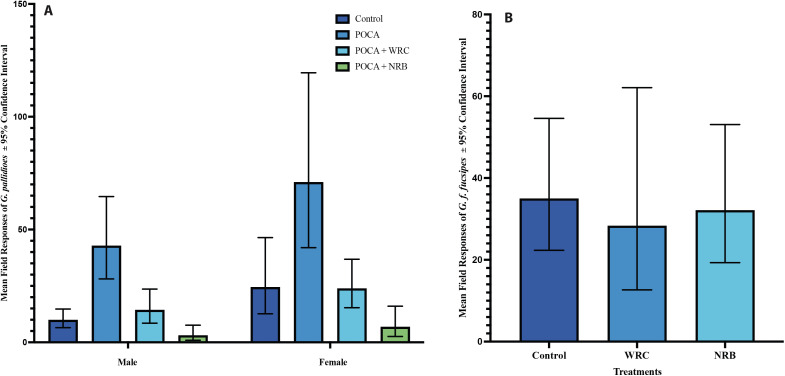
Geometric mean catches of *G. pallidipes* and *G. f. fuscipes* in traps and targets with various odor treatments in Shimba Hills National Reserve and Ndere National Park respectively, in Kenya.

We have summarized the data on abundance of arthropods captured in the traps and targets in Shimba Hills National Reserve and Ndere Island National Park in Fig 1. Tsetse flies were most abundant, constituting 97 and 64% of arthropods catches in the reserve and park, respectively. Among the tsetse flies, *G. pallidipes*, *G. austeni* and *G. brevipalpis* were caught in traps in Shimba Hills National Reserve, where *G. pallidipes* was the most abundant, while only *G*. *f. fuscipes* were captured in targets in Ndere Island National Park. The pattern of catches between male and female *G. pallidipes* to the treatments was significantly different (*X*^2^_(3, *N* = 8)_ = 17.12, *p* < 0.001). On the other hand, pattern of catches between sexes in *G. f. fuscipes* responses to the treatments were similar (*X*^2^_(2, *N* = 6)_ = 2.70, *p* = 0.2594). Consequently, ANOVA for male and female *G. pallidipes* responses to treatments were conducted separately, whereas data on responses of male and female *G. f. fuscipes* to the treatments were pooled for the analyses. Overall, indices of responses of male *G. pallidipes* to WRC and NRB, relative to no-odor control, were 1.4 and 0.3 respectively, while the indices for responses of the females to the same were 1.0 and 0.3 respectively (S1 Table). On the other hand, indices of responses of *G. f. fuscipes* to WRC and NRB were 0.8 and 0.9 respectively (S1 Table). Consequently, both sexes of *G. pallidipes* were most and least attracted to traps baited with POCA and POCA+NRB blends respectively (Fig 2A). Responses of both sexes of *G. pallidipes* in the traps baited with WRC and control traps (without odor) were similar (*p* > 0.05). However, *G. pallidipes* responses to traps baited with NRB were significantly less than those responding to traps baited with WRC, where male and female *G. pallidipes’* responses to traps baited with NRB were 2.0 (F_(3, 47)_ = 18.8015, p < 0.001) and 1.6 (F_(3, 47)_ = 12.0391, p < 0.001) folds less respectively, than the flies responding to WRC (Fig 2A, S1 Table). Responses of *G. f. fuscipes* to the traps with WRC, NRB or control (un-baited) were similar (F _(2, 26)_ = 0.1227, p = 0.8855) (Fig 2B, S1 Table).

## Discussion

Humans and their livestock can be protected from infectious and/or nuisance bites from arthropod by avoiding arthropod habitats or by using arthropod behavior modifying repellents active against target arthropod(s). Repellent technologies constitute a fundamental approach for prevention of transmission of arthropod-borne diseases, with spatial repellents (spatial emanators) augmenting contact repellents and pesticides within an integrated vector management framework at minimal cost. The spatial repellents can also be deployed alone to potentially protect humans and their livestock from the vectors, especially in settings with limited coverage by other vector control interventions. Most spatial repellents have been developed for and deployed against mosquito-borne diseases [29]. Development of spatial repellents against tsetse flies is fairly recent, with only Waterbuck Repellent Compound (WRC) [15] and Novel Repellent Blend (NRB) [11] being described to date for potential protection of humans and their livestock. Our findings in the present study reveal 3.5 and 4.7 -folds better repellence (mean indices of fly catches in WRC relative to NRB baited traps) of female and male *G. pallidipes,* respectively, by NRB relative to WRC. These remarkable observations in behavioral responses of the flies may be due to differences in their olfactory perception of the repellents, suggesting that NRB can potentially provide enhanced protection of humans and their livestock from *G. pallidipes* bites and associated trypanosomiasis (HAT/AAT) transmission and nuisance. The WRC exhibits >80% efficacy in protection of cattle against *G. pallidipes* [15]. Consequently, NRB is expected to exhibit near absolute efficacy against *G. pallidipes*, based on the current comparative performance against WRC, which should be empirically established. The absence of efficacy of either repellents against *G. f. fuscipes*, suggests that NRB and WRC may have a narrow range of tsetse fly species specificity and hence necessitate a need for expanded search for repellents active against *G. f. fuscipes*. Additionally, extent of efficacy or lack thereof of the repellents within morsitans/savannah and palpalis/riverine group, of flies, to which *G. pallidipes* and *G. f. fuscipes* respectively belong remains to be determined. Such group-wide responses have been observed in relative efficacy of POCA attractant against morsitans and palpalis, with POCA being effective and ineffective against the former and latter, respectively [17,18,30]. Additionally, these findings suggest that protection of vertebrate hosts by either repellents would be sufficient or partial in the field, where *G. pallidipes* is the only species or sympatric with *G. f. fuscipes*, respectively. Unfortunately, *G. pallidipes* is sympatric with other savannah species and/or palpalis group of tsetse flies in most of their habitats in sub-Sahara Africa, suggesting a need to: 1) assess efficacy of either repellents against other tsetse fly species such as *Glossina austeni*, *Glossina swynnertoni*, *Glossina morsitans centralis* that are typically sympatric with *G. pallidipes*, or 2) prospect for and formulate effective novel repellents against *G. f. fuscipes.*

Effective administration of the NRB for human and livestock protection will require development and optimization of a novel, needs specific, delivery system for the repellent. The current delivery system employs sealed thin-walled polythene sachets constructed from polyethylene lay-flat tubing adapted from delivery systems designed for dispensation of POC components of POCA [31] on stationary traps and targets. The NRB on the other hand should ideally be dispensed from moving subjects (hosts) and at a rate sufficiently controlled to extend the utility/longevity of the product. The waterbuck-like smell associated with these repellents can also be masked with a pleasant smell to enhance acceptance for use within proximity of vertebrates, including humans. The repellent can potentially be integrated into the woven fabric and nets to broaden its utility. So far, NRB has successfully been micro-encapsulated in beta cyclodextrin nano-particles to moderate release of the repellent [32]. However, field performance of the encapsulated product in repellence of the flies remains to be determined.

In conclusion, NRB appears to be several-fold more repellent than WRC against *G. pallidipes*. The impact of this enhanced repellence on livestock protection remains to be determined. Both NRB and WRC are ineffective against *G. f. fuscipes,* suggesting possible tsetse fly species specificity among the repellents and the need to determine a spectrum of tsetse fly species against which these repellents are efficacious. Development and optimization of a novel delivery system for NRB can partially expand its utility and application in vector control.

## Supporting information

S1 Table Analysis of variance of responses of *G. pallidipes* and *G. f. fuscipes* to various odor treatments in Shimba Hills National Reserve and Ndere National Park respectively, in Kenya. (XLSX)
